# Rehabilitation and Long Term Outcomes Including Return to Work or Sport Following Reverse Total Shoulder Arthroplasty

**DOI:** 10.1007/s12178-025-09948-9

**Published:** 2025-02-07

**Authors:** Christopher J. Lamb, Aamir Ahmad, Brett M. Biedermann, Eric H. Lin, Jacob L. Kotlier, Christian A. Cruz, Frank A. Petrigliano, Joseph N. Liu

**Affiliations:** Department of Orthopaedic Surgery, Keck School of Medicine of USC, 1520 San Pablo St #2000, Los Angeles, CA 90033 USA

**Keywords:** Reverse total shoulder arthroplasty, Return-to-sport, Return-to-work

## Abstract

**Purpose of Review:**

Reverse total shoulder arthroplasty has become an excellent surgical option for patients suffering from various shoulder pathologies including rotator cuff arthropathy, inflammatory arthritis and proximal humerus fractures. The goals of this operation are to reduce pain, restore function, and allow patients to be able to return to both work and sport. This article provides insight into the return to work and sport of patients who have undergone reverse total shoulder arthroplasty.

**Recent Findings:**

Recent literature has demonstrated that patients who have undergone reverse total shoulder arthroplasty demonstrate high rates of return to work and sport. Variations in patient age, sex, work intensity, type of sport and rehabilitation protocols can also play a factor in being able to return to work and sport.

**Summary:**

Patients who have undergone reverse total shoulder arthroplasty are able to return to work and sport in a timely manner. A structured rehabilitation protocol, appropriate patient selection and excellent communication between surgeon and patient is crucial to achieve a successful return to work and sport.

## Introduction

Advancements in reverse total shoulder arthroplasty (rTSA) has transformed shoulder reconstruction surgery. In the past 20 years, there has been a significant increase in utilization of rTSA [1]. rTSA is now used in multiple settings including rotator cuff arthropathy, fracture care, inflammatory arthropathies and revision cases [2–5]. In a normal shoulder joint, the rotator cuff acts as a compressive force on the humeral head to preserve the center of rotation. rTSA functions by reversing the normal ball and socket configuration of the shoulder joint. The glenosphere ball is placed in the socket and the socket is placed in the position of the humeral head [6]. This prosthesis design creates a semi-constrained construct and stabilizes the glenohumeral joint by medialization and distilization of the center of rotation. This medialization and distilization of the center of rotation allows for optimization of the deltoid lever arm and the deltoid muscle is lengthened and is able to compensate for rotator cuff deficiency [7]. rTSA has demonstrated excellent clinical outcomes in both medium and long-term follow up [8, 9]. Patients undergoing rTSA are able to obtain activity levels equivalent to patients undergoing conventional anatomic total shoulder arthroplasty and humeral hemiarthroplasty [10, 11].

There have been several studies that have investigated return to work and sport after shoulder arthroplasty. Previous studies have suggested that patients return to work around 2.3 months post-operatively after shoulder arthroplasty [12]. The goal of this systematic review and meta-analysis was to investigate return to work and sport after reverse total shoulder arthroplasty.

## Rehabilitation

Rehabilitation stands as a critical aspect of the postoperative care for individuals undergoing reverse total shoulder arthroplasty. Postoperatively, patients may experience pain, reduced range of motion (ROM), and strength deficits. Existing literature has consistently demonstrated significant improvements in ROM, pain scores, patient-reported outcomes, and overall patient satisfaction. One study highlighted continuous enhancements in ROM and the Oxford Shoulder Score for up to 1 year postoperatively, underscoring the importance of long-term rehabilitation after rTSA [13].

While the significance of rehabilitation in rTSA recovery is acknowledged, there exists considerable heterogeneity among rehabilitation protocols. A systematic review conducted by Bullock et al. revealed a diverse array of proposed protocols for immobilization and passive ROM, stemming from considerations of biomechanical properties, healing time frames, and exercise loading principles [14]. Despite this diversity, a common ground emerged among the included studies, emphasizing the necessity of early deltoid strengthening due to the altered anatomy of the shoulder joint. This consensus is further supported by another study highlighting the importance of the deltoid and periscapular muscles in rTSA rehabilitation, although minimal consensus exists regarding the integration of sling immobilization and early passive ROM [15].

Despite the observed heterogeneity, the consensus regarding the integral role of rehabilitation in rTSA recovery and future function remains consistent throughout the literature. Randomized controlled trials comparing early versus delayed mobilization and rehabilitation have consistently demonstrated significant improvements in both groups, with no significant long-term differences between them [16, 17]. Another retrospective cohort study, categorizing patients into groups with varying durations of immobilization (none, 3 weeks, and 6 weeks), found no differences in pain, patient-related outcomes, ROM, and patient satisfaction at various intervals up to 1 year [18].

Despite minimal disparities in short- and long-term outcomes with various rehabilitation protocols, concerns persist regarding early mobilization. Some studies discuss complications of early mobilization, such as acromion and scapular spine fractures. The biomechanical and anatomical reasons for these fractures, potentially linked to increased deltoid stress on the acromion, are subjects of debate [19]. Considering other patient factors is crucial in determining the correct rehabilitation protocol. Early mobilization may be beneficial for the elderly population to avoid limiting function and increasing fall risk post-recovery. Various factors, including home versus supervised therapy, preoperative conditioning, and patient adherence, necessitate further research. A tailored rehabilitation protocol for rTSA is essential for improved recovery, and further large cohort studies should be conducted to determine the ideal protocol for maximizing ROM, strength, and long-term outcomes while minimizing pain.

### Long-term Outcomes

With rTSA being a relatively recent procedure, an expanding body of literature addresses its long-term outcomes (Table 1). Initial literature suggested improvements in mid-term follow-up with a subsequent decline in performance during long-term follow-up. One study reported significant decreases in Constant scores from mid-term to long-term follow-up (3.3 to 12.5 years of follow-up) [20], while another indicated decreased Constant scores for patients with greater than 7 years of follow-up compared to < 5 years [21]. However, recent studies have shifted this narrative, demonstrating that excellent short-term outcomes, such as improved forward flexion and American Shoulder and Elbow Surgeons (ASES) scores, are maintained through long-term follow-up [22–24]. This sustained improvement in function suggests a potential definitive treatment option through the end of life for patients with arthritic glenohumeral joints.

**Table 1:** Postoperative outcomes of reverse total shoulder arthroplasty

Study	Population	Rehabilitation Protocol	Outcomes
Lee et al. (2021) [18]	357 rTSA in 320 patients (3 groups: no immobilization, 3 weeks, and 6 weeks immobilization)	• Varying length of initial immobilization • Passive ROM • Deltoid strengthening • Supplementary strengthening of scapular stabilizers and proprioceptive rehab	Each group improved in ROM, pain, and PRO scores. No significant difference was found between groups
Edwards et al. (2021) [16]	63 rTSA in 61 patients (2 groups: early AAROM at 2 weeks, delayed AAROM at 6 weeks)	• Shoulder immobilization for 6 weeks • Delayed group: 2–6 weeks PROM, 6–12 weeks AAROM, 12–20 weeks activity as tolerated • Early group: 2–6 weeks PROM and AAROM, 6–12 weeks AROM, 12–20 weeks active strengthening	Early active group reported improved shoulder flexion at 3 months and PROs between 3–6 months. No significant difference between groups was seen at 3-, 6-, or 12-months post-surgery
Hagen et al. (2020) [17]	86 rTSA (2 groups: early ROM, 6-week delayed ROM)	• Delayed group: sling immobilization with no PROM or AROM for 6 weeks • Early group: immediate rehabilitation with PROM and AROM • Neither group had strengthening exercises until 6 weeks	Both groups showed significant improvements in ROM and ASES scores. No significant differences were found between groups except improved ASES scores in the delayed group at 6 weeks
Kornuijt et al. (2023) [13]	100 rTSA (1 group: early active rehab)	• 0–1 week: PROM • 2–4 weeks: PROM, AAROM, isometric deltoid exercises • 4–6 weeks: Progressive strengthening, scapular stabilization, rotator cuff strengthening if present • 7–12 weeks: Progression of previous exercises, ADL training • > 12 weeks: Optimize ROM and strength, return to work, sports, and ADLs	Direct rehab may be safe and effective. A potential 5% rehab complication rate was noted

*ROM* range of motion, *PRO* patient related outcome, *AAROM* active assisted range of motion, *AROM* active range of motion, *PROM* passive range of motion, *ADL* activity of daily living, *ASES* American Shoulder and Elbow Surgeons

The sustained long-term benefits observed may be attributed to improvements in implant design. A study examining the use of a standard cemented long stem rTSA found increased forward elevation, abduction, and patient satisfaction with decreased pain after a 10-year follow-up [25]. Growing interest in stemless rTSA components due to humeral stem-related complications has been noted. A systematic review found similar results between stemmed and stemless components, with decreased humeral stem complications in the stemless studies [26].

While most current literature involves patients over the age of 60, a study aimed to determine mid-term outcomes in a younger population (< 60) and found similar outcomes of improved ASES and active forward flexion [27]. However, patients with higher-demand occupations exhibited less improvement in pain scores [27]. Patient-specific factors should be considered when determining surgical indications and the risk of future complications, especially in younger or more active patients. Common complication of rTSA reported is instability associated with dislocation, stem loosening, scapular notching, and revision [23, 25]. Further research is warranted to determine the effects of age, various comorbidities, and implant design on the long-term outcomes of rTSA.

## Return to Sport

Studies which examine rates of return to sport (RTS) after rTSA report widely varying statistics (Table 2). A retrospective review of athletes who underwent reverse total shoulder arthroplasty between 2017 and 2020 estimated the rate of RTS as 63.6% [28]. However, other studies report rates as high as 83% [29]. A systematic review on return to sport in 457 patients across six studies found that RTS rates varied from 60 to 93%, with an overall average of 79% [30]. While the specific rates of return vary between studies, most studies agree that the majority of patients are able to successfully return after surgery.

**Table 2:** Studies examining return to sport after reverse total shoulder arthroplasty

Study	Study Type	Age (years)	Procedure	Rate of Return	Time to Return (mo)	Other Findings
Al Housni 2022 [34]	Prospective	74 (range 51–88)	106 rTSA	67% at 6 months and 48% at 4 years	NS	Younger age and male sex were found to have a significant positive correlation with RTS
Franceschetti 2021 [30]	Systematic review	74.7 (range 33–88)	457 rTSA	79%, (range 60%−93%)	5.3	Average patient satisfaction for rTSA was 88.3%
Kim 2023 [32]	Retrospective	72.4 ± 4.9 for RTS and 73.7 ± 7.1 for no RTS	44 rTSA	63.60%	mean 9.1, (range 3–36)	The group of athletes who did not RTS had a statistically significant higher BMI when compared to those who did RTS
Küffer 2021 [31]	Systematic review	68 (range 18–92.6)	928 rTSA and 814 aTSA	75% for rTSA and 77.4% for aTSA	7.9 (range 1.5–36) for aTSA and 6.2 (range 1–36) for rTSA	For rTSA, mean RTS at a better level was 40%, but at the same level was 77%. The mean RTS at a worse level of sport was 10.6%
Papalia 2020 [33]	Meta-Analysis	72.5	375 aTSA and 750 rTSA	77% for rTSA and 90% for aTSA	mean 7, (range 5.3–11)	The most practiced sports after surgery are low contact activities such as fitness, swimming, golf, and tennis
Tangtiphaiboontana 2021 [35]	Retrospective	70 (range 34–86)	109 rTSA	70.10%	NS	Activities with the highest return rates were sailing (92%), fishing (91%), dancing (88%), weight training (88%), hiking (86%), elliptical/treadmill (86%), and calisthenics (86%)
Tramer 2021 [40]	Retrospective	69.6 ± 8.9	14 rTSA and 17 aTSA	74%	NS	The RTSA group had a significantly lower postoperative driving distance compared to the TSA group
Shimada 2023 [29]	Retrospective	72 ± 6 for rTSA and 71 ± 8 for aTSA	60 rTSA and 30 aTSA	83% for rTSA and 93% for aTSA	NS	One ATSA shoulder showed loosening of the glenoid implant, and 8 RSA shoulders demonstrated low-grade scapular notching

*aTSA* anatomic total shoulder arthroplasty, *NS* not specified, *rTSA* reverse total shoulder arthroplasty, *RTS* return to sport

Aside from rate of return, the amount of time required to fully return to preoperative levels of activity is also critical. The amount of time an athlete must refrain from activity interferes with training and makes recovery to preoperative fitness levels more difficult. Küffer et al. recently conducted a systematic review of 2199 athletes receiving either aTSA (anatomic TSA), rTSA, or hemiarthroplasty [31]. In this review, the mean time to return to sport was 7.9 (range 1.5–36.0) months after aTSA and 6.2 (range 1–36) months after rTSA. Similar results were demonstrated in a meta-analysis reporting an average 7 (range 5.3–11) months after rTSA. A recently performed retrospective study gave a longer time estimate of 9.1 (range 3–36) months [32].

Return to sport rates for rTSA appear to be lower when compared to aTSA. A meta-analysis on 11 studies reported a return to sport rate of 90% after anatomic TSA and a rate of 77% after reverse TSA [33]. Additional studies supported this pattern. In a 2023 study including both athletes and workers, Shimada et al. reported a 93% return rate after anatomic TSA compared to 83% after reverse TSA [29]. Additionally, postoperative external and internal rotations, Constant scores, and satisfaction were significantly higher in aTSA compared to rTSA. The superior clinical outcomes observed after aTSA may explain why the rate of return is higher when compared to rTSA. Despite this apparent difference, the overall rate of return is still high for both procedures.

Beyond the procedure used, multiple other factors contribute to the wide variance in rates of return to sport after rTSA. Kim et al. explored the effect of activity load, where complete return to sport for low load activities was 67%, return for medium load was 24% and high load was 50% [32]. This study also found a significantly lower BMI in the RTS group vs the non RTS group. In other studies, factors identified to influence RTS rates include sex, age, sport type, and preoperative range of motion and strength [34]. The effect of sport type has been further investigated in studies. Papalia et al. listed the types of activities patients most commonly return to. In this study, swimming had the highest rate of return (84%), followed by general fitness (77%), golf (77%), and tennis (69%) [33]. A subsequent study performed a similar analysis and found the highest RTS rate for sailing (92%) followed by fishing (91%), dancing (88%), weight training (88%), hiking (86%), elliptical/treadmill (86%), and calisthenics (86%) [35]. The specific sports listed vary between the two studies, possibly due to differences in the demographics and preferences of the included populations. Still, there is a consistent pattern of non-contact and lower demand sports resulting in the highest return rates.

A 2021 study surveyed 109 athletes who underwent a rTSA and asked participants about their reasons for not returning [35]. Patients who did not return reported restricted motion (22%), fear of reinjury (21%), and weakness (18%) as the most common reasons for not returning to their sport [35]. Most studies on RTS focus on the association between predetermined factors and RTS rates, but it would also be valuable to have more studies with open ended questions where participants can explain why they did not RTS. Athletes may self-report reasons that differ from the factors identified by researchers. Further, an athlete’s assessment of their own ability after recovering from surgery may differ from a clinician’s objective measurements, while still influencing whether or not they return to sport. For example, Tangtiphaiboontana et al.’s finding of athletes describing a “fear of reinjury” suggests a role of psychological factors in the decision to return [35]. Specifically, kinesophobia, or a fear of movement after injury, has been studied as a relevant postoperative measure and has been correlated with RTS after shoulder stabilization surgery. [36, 37] The role of psychological readiness in RTS is a new area of research showing promising results in studies on shoulder and knee injuries. For instance, a recent meta-analysis analyzed RTS following ACL tears, demonstrating that athletes who RTS have higher psychological readiness scores [38]. Given the findings of these studies, future research would benefit from further investigating the role of psychological readiness in the context of shoulder arthroplasty.

## Return to Work

Most patients are able to RTW (return to work) after recovering from rTSA (Table 3). Estimates of the RTW rate vary from 56 to 65% in the literature [39]. A recent systematic review including 447 patients calculated an overall 61.5% return to work rate following reverse shoulder arthroplasty surgery [10]. Returning to work can be defined in two different ways: either a return to full preoperative duties or a return to work at any level, including with a decreased workload. Jayasekara et al. characterized both categories in a retrospective study [28]. Of the 34 rTSA patients, 19 (56%) returned to any level of work by the 6 month follow up. Eight (23%) of these patients returned to work at full duties while eleven (32%) returned to lighter duties [28].

**Table 3:** Studies examining return to work after reverse total shoulder arthroplasty

Study	Study Type	Age (years)	Procedure	Rate of Return	Time to Return (mo)	Other Findings
Jayasekara 2020 [28]	Retrospective	55 (range 12–91)	34 rTSA and 38 aTSA	56% for rTSA and 71% for aTSA	NS	The best independent predictors of RTW were younger age, less stiffness, and working preoperatively
Lalehzarian 2022 [39]	Systematic review	NS	rTSA and aTSA	(range 56%−65%) for rTSA and (range 71%−93%) for aTSA	2.3 ± 2.4 rTSA and 1.93 ± 3.74 for aTSA	Workers’ compensation status negatively influenced clinical outcomes following shoulder arthroplasty
Steinhaus 2019 [10]	Systematic review	63.6 range (27.6–97.1)	65 rTSA and 122 aTSA	65.3% for aTSA and 61.5% for rTSA	mean 2.3, (range 0.3–24)	RTW was significantly lower for patients with heavy-intensity occupations. RTW did not differ between rTSA, aTSA, or hemiarthroplasty

*aTSA* anatomic total shoulder arthroplasty, *NS* not specified, *rTSA* reverse total shoulder arthroplasty, *RTS* return to sport

The amount of time required to recover after surgery is also an important consideration for patients considering rTSA, as time spent off from work directly affects patients’ income. One review estimated a mean time to RTW of 2.3 months, with a range of 0.3 to 24 months after rTSA [10]. A subsequent meta-analysis agreed with these estimates. In data collected from 12 studies, patients returned to work an average of 2.3 ± 2.4 months after rTSA and 1.93 ± 3.74 months after aTSA. Notably, this study also found that workers’ compensation status negatively influenced clinical outcomes following shoulder arthroplasty [39]. In comparison to studies focused on athletes, time to return to activity is quicker for workers than for athletes.

Consistent with the findings seen in studies on athletes, the return to work rate appears to be higher after aTSA compared to rTSA. A recent study examined outcomes of 12 different shoulder surgeries, and rTSA had the lowest RTW rate of the procedures. rTSA had a 56% RTW rate while aTSA had a 71% return rate [28]. Multiple other studies support this pattern, with RTW estimates ranging from 56%−65% for rTSA and 71%−93% for aTSA. As discussed previously, the time to return has also been found to be longer in rTSA compared to aTSA [39]. However, this consensus is not without debate. A systematic review challenged these results by finding no difference in RTW between anatomic TSA, rTSA, or hemiarthroplasty [10]. It is worth noting that this study was limited by a small sample size and heterogenous data**.** Additionally, the authors proposed the potential explanation that patients receiving hemiarthroplasty tend to be younger, and therefore may be more likely to return to work. Studies have also found that rTSA patients may be older than aTSA patients [40]. There are likely confounding factors which influence both the decision of what procedure to perform as well as ability to return to work after recovery.

Multiple factors have been identified which influence RTW rates. A study of 1773 employed patients examined factors influencing RTW after shoulder surgery [28]. This study demonstrated that intensity of work (light, moderate, or strenuous) had the highest correlation with RTW. Other factors which showed a significant correlation with return rates were age at operation, sports activity level, shoulder stiffness, and patients’ perception of shoulder. Similarly, another meta-analysis concluded that heavy-intensity vs light-intensity occupations as the primary factor influencing return to work across seven studies [10]. While these two studies include data from multiple different shoulder surgeries, it is likely that the factors affecting return rates for rTSA would be similar to other shoulder arthroplasties. Still, there is room for future studies which examine more detailed factors affecting RTW specifically in the context of rTSA.

For both return to work and return to sport, overall rates of return after rTSA are high; however, rates vary by study due to the influence of various factors such as sex, age, sport type, and preoperative range of motion and strength. Psychological factors may also influence return rates. More research is needed to characterize these factors further, as it is important to take them into account when creating guidelines for return to sport/work. Further, these are important considerations for clinicians evaluating individual patients on returning to sport or work after reverse total shoulder arthroplasty.

Despite the promising results, it is important to know many studies in this area of research share common limitations. Most systematic reviews in this topic rely on studies with a high level of heterogeneity, due to the involvement of different surgeons, different patient demographics and activity levels, and other factors. The definition of RTS/RTW also varies in the literature. Studies may define return as being able to return to work/sport at any level, or they may only consider a complete return to preinjury levels of activity. Most studies are retrospective and of lower levels of evidence; there is a lack of high-level prospective trials studying return to work or sport in the context of rTSA. Patient satisfaction and ability to return is multifactorial, depending on age, sex, sport/occupation type and range of motion; therefore, surgeons should engage in preoperative discussion with the patient to set expectations on return to work/sport.

### Authors Recommendation

We recommend beginning rehabilitation within the first week following shoulder arthroplasty under the guidance of a physical therapist, with therapy typically lasting for 3 to 6 months. For returning to work, patients can generally resume sedentary work within 1 to 2 months, light work around 2 to 3 months, moderate work within 3 to 6 months, and heavy work after 6 months or more, depending on individual recovery. Regarding sports, low-impact activities such as swimming and cycling can usually be resumed within 4 to 6 months post-operatively. High-impact sports may be resumed cautiously within 6 to 8 months, though participation in contact sports is generally not recommended. For long-term activity, we advise considering restrictions, particularly for high-impact and contact sports, as these may accelerate component wear. Ultimately, each patient should be individually assessed to determine their ability to perform specific tasks, and long-term work or activity restrictions should be tailored to their functional capacity and clinical needs. These recommendations aim to optimize recovery and ensure a safe return to daily activities, work, and sports, tailored to the unique circumstances of each patient.

## Conclusions

The majority of patients return to sport after rTSA, with estimates of RTS rates varying between 60 to 93%. In regards to return to work, estimates vary from 56 to 65%. rTSA has been shown to provide improvements in ROM, pain scores, patient-reported outcomes, and overall patient satisfaction. rTSA is indicated for irreparable rotator cuff tear/rotator cuff arthropathy, proximal humerus fractures, inflammatory arthritis and failed prior shoulder arthroplasty. Contraindications include complete axillary nerve palsy, deltoid dysfunction, active infection, glenoid bone stock deficiency and neuroarthropathy. Common complications include instability associated with dislocation, stem loosening, scapular notching, and the need for revision. A proper rehabilitation protocol should include early deltoid strengthening due to the altered anatomy of the shoulder joint. Early versus delayed mobilization protocols both have similar outcomes; however, there is concern with increased complications in early mobilization. The success of recovery is primarily influenced by the intensity of activity (either of the sport or of the work); however, sport type, age, sex, BMI, and worker's compensation status also influence recovery. Psychological factors, such as a fear of reinjury, may also play a role in athletes.

## Data Availability

No datasets were generated or analysed during the current study.
